# The value of signs, symptoms and plasma heart-type fatty acid-binding protein (H-FABP) in evaluating patients presenting with symptoms possibly matching acute coronary syndrome: background and methods of a diagnostic study in primary care

**DOI:** 10.1186/s12875-014-0203-8

**Published:** 2014-12-12

**Authors:** Robert TA Willemsen, Frank Buntinx, Bjorn Winkens, Jan F Glatz, Geert Jan Dinant, The ‘RAPIDA’-study team (‘RAPIDA’: RAPid test for Investigating complaints possibly Due to Acute coronary syndrome)

**Affiliations:** Department of General Practice, Maastricht University, PO box 616, Maastricht, 6200 MD Netherland; Department of General Practice, Catholic University (KU) Leuven, Kapucijnenvoer 33 blok j bus 7001, Leuven, B-3000 Belgium; Department of Methodology and Statistics, Maastricht University, PO box 616, Maastricht, 6200 MD Netherland; Department of Molecular Life Sciences, Maastricht University, PO box 616, Maastricht, 6200 MD Netherland

**Keywords:** Acute coronary syndrome (ACS), Acute myocardial infarction (AMI), Diagnostic study, Heart-type fatty acid-binding protein (H-FABP), Point of care test (PoCT), Primary care

## Abstract

**Background:**

Chest complaints presented to a general practitioner (GP) are frequently caused by diseases which have advantageous outcomes. However, in some cases, acute coronary syndrome (ACS) is present (1.5-22% of cases). The patient’s signs, symptoms and electrocardiography results are insufficient diagnostic tools to distinguish mild disease from ACS. Therefore, most patients presenting chest complaints are referred to secondary care facilities where ACS is then ruled out in a majority of patients (78%). Recently, a point of care test for heart-type fatty acid-binding protein (H-FABP) using a low cut-off value between positive and negative of 4 ng/ml has become available. We aim to study the role of this point of care device in triage of patients presenting chest complaints possibly due to ACS, in primary care. Our research protocol is presented in this article. Results are expected in 2015.

**Methods/Design:**

Participating GPs will register signs and symptoms in all patients presenting chest complaints possibly due to ACS. Point of care H-FABP testing will also be performed. Our study will be a derivation study to identify signs and symptoms that, combined with point of care H-FABP testing, can be part of an algorithm to either confirm or rule out ACS. The diagnostic value for ACS of this algorithm in general practice will be determined.

**Discussion:**

A safe diagnostic elimination of ACS by application of the algorithm can be of significant clinical relevance. Improved triage and thus reduction of the number of patients with chest complaints without underlying ACS, that are referred to secondary care facilities, could lead to a substantial cost reduction.

**Trial registration:**

ClinicalTrials.gov, NCT01826994, accepted April 8^th^ 2013.

## Background

### Daily practice

Patients presenting with chest complaints caused by acute coronary syndrome (ACS) need urgent transport to a specialist setting [1,2]. In these situations, favourable outcome is inversely related to the time interval between onset of complaints and vascular rescue treatment. However, in daily practice, most patients experiencing chest complaints primarily apply to a general practitioner (GP). Within this population, benign conditions such as thoracic wall complaints, gastric disease or psychiatric and somatoform disease, largely outnumber cases of ACS [3-5]. In specialised care facilities such as coronary care units, 50% of patients presenting with chest pain are diagnosed with ACS, whereas in primary care, ACS is diagnosed in no more than 1.5-22% of cases [6-8]. Referring every patient with chest complaints would overwhelm secondary care facilities, however the GP is faced with serious diagnostic dilemmas since milder diseases with beneficial outcome can mimic ACS and vice versa [9]. Safe exclusion, rather than inclusion, is the main task of a GP in assessing chest complaints.

In primary care, where clearly circumscribed diseases with specific symptoms are embedded in a broad scope of illness and mild disease with less typical appearance, discriminative signs are usually scarce. This applies in particular to ACS, where history, physical examination and electrocardiography notoriously lack sensitivity and specificity as has been extensively surveyed [10-12]. With regard to chest pain, only few signs and symptoms, for example, the absence of pain on chest palpation or presence of pain on exertion, generate discriminative strength in diagnostic studies, although to a limited extent [9,13,14]. Altogether, clinical signs and symptoms are inappropriate for ruling out ACS or lack additive value above the clinical judgment by the GP, which on itself is correct in a majority of cases [6]. Weighing more abstract factors (e.g. contextual patient factors and altered presentation in time) probably leads to this fairly adequate clinical judgment of GPs [15-17].

Because of these diagnostic problems in primary care, most patients presenting complaints possibly matching ACS are still referred to a cardiologist [18]. A low threshold for referring patients with chest complaints to secondary care is an effective strategy for the GP to avert missed cases of ACS [19]. In the Netherlands, this strategy results in 78% of referred patients that are ACS negative in the emergency room (positive predictive value (PPV) is 22%), whereas ACS is present in 5% of patients that are not referred (negative predictive value (NPV) is 95%) and the outcome is probably not severely affected by initially not referring (specificity and sensitivity of clinical assessment by GP: 55%, resp 81%) [6,20]. To enhance triage by a GP and reduce patient burden in secondary care, new diagnostic tools should become available [21]. Combined with signs and symptoms, such tools should be able to safely rule out ACS in a significant number of otherwise referred patients, without a rise in missed cases of ACS. Importantly, this would lead to a significant cost reduction. The number of referred patients would decrease and in the remaining patients who are referred, ACS could be confirmed or ruled out as common in secondary care.

In the field of pulmonary embolism and respiratory tract infections, diagnostic tools combining clinical signs and symptoms with the result of a point of care (PoC) test have recently been introduced. Both increased efficiency by either reducing unnecessary referral (in cases of suspected pulmonary embolism) or treatment (in respiratory tract infections) [22-24]. For ACS, a similar procedure has not yet been defined.

### Biomarkers and point of care testing in primary care

Cardiac troponin T or I (cTnT or cTnI) measurement has become the cornerstone of diagnosing acute myocardial infarction (AMI) [25-29]. Some promising novel biomarkers have featured in recent literature, for example, copeptin and heart-type fatty acid-binding protein (H-FABP) [30-32]. Adding measurements of these markers to troponin in an early phase in emergency room settings increases sensitivity for ACS, but so far the combination has failed to safely rule out ACS in an early stage [33,34]. Until recently, troponin assays have gained sensitivity due to usage of highly sensitive techniques, referred to as high sensitive cardiac troponin (hs-cTn). The additional value of H-FABP testing besides hs-cTn is unclear [35,36]. At this moment, studies reviewing early PoC markers are characterised by methodological imperfections [37]. The function of H-FABP and other early markers combined with signs and symptoms in risk classification in a low prevalence setting such as primary care is still to be determined [38-40].

An earlier study in primary care evaluating a PoC-test on H-FABP did not lead to implementation of PoC-testing in daily practice because of a lack of NPV [20]. However, the PoC device for H-FABP used in this study used a cut-off value of 7 ng/ml, which is above the 99^th^ percentile of 5.7 ng/ml as found in a normal reference population [41]. Retrospective measurement of plasma H-FABP values revealed added value of H-FABP, although insufficient to reach a NPV of 98% or more. Recently, a potential gain in sensitivity for PoC-testing has been found by lowering the cut-off value to 4 ng/ml in a secondary care population, where 50% of patients were diagnosed with AMI [8]. Setting the cut-off value at 4 ng/ml leads to a diagnostic performance equalling that of hs-cTn. Calculated NPV in a primary care population (with an incidence of ACS of 20% or less) would reach 88.3% in patients with a duration of complaints of less than 3 hours and 97.9% in patients with a duration of complaints of 3–24 hours.

When H-FABP-testing is combined with signs and symptoms in a *diagnostic algorithm*, NPV hypothetically improves and thus the number of patients that are referred by a GP but turn out to have no ACS, could be reduced. Even with a moderate amount of false positive results, such an algorithm could improve daily practice since currently, the majority of patients without underlying ACS are referred to secondary care facilities. Thus, our hypothesis is that net reduction of referral rate – without an increase in missed cases of ACS – in patients presenting chest complaints to their GP could be accomplished by the aforementioned algorithm including PoC H-FABP-testing. Therefore a PoC H-FABP-device meeting the demand of a lower cut-off value of 4 ng/ml has recently been developed by FABPulous B.V. We intend to study the diagnostic value of this device in primary care. The methodology of the study is presented in the remainder of this article in order to inform colleagues in the same research field, to anticipate forthcoming results of our study and to prevent publication bias or violation of the protocol during the running time of the study.

### Study design and objectives

This current study is a derivation study to identify signs and symptoms that, combined with point of care heart-type fatty acid-binding protein (PoC H-FABP) testing, can be part of an algorithm to confirm or rule out ACS (meaning AMI and unstable angina (UA)) and AMI alone in a population of patients with complaints possibly due to myocardial ischaemia in general practice. Thus, stage 2 of the 6 major stages in the development and testing of a clinical decision rule as defined by Stiell and Wells will be studied [42]. The diagnostic value of the algorithm will be studied and compared to regular assessment by a GP without PoC H-FABP testing using a delayed type cross-sectional diagnostic model. Both ACS and AMI as a subgroup of ACS will be analysed as final diagnosis. The cost-effectiveness of the test will also be evaluated. The study commenced in November 2013 and will run for two years. External validation of the algorithm derived from this study needs to be performed in future studies.

## Methods

### Recruitment

Two hundred and ten GPs in four regions in the South and Southeast of the Netherlands and the Northeast of Belgium, including both urban and rural areas, will be informed by e-mail of our study and given the opportunity to respond. The first 60 GPs that decide to participate will receive a detailed training in using the test and performing all relevant research activities in order to include ten successive eligible patients, making a total of 600 patients. Inclusion will take place at any time during the day and night, including either during consultation in a primary care facility or during home visits by participating GPs. During evaluations in the study period with participating GP’s, adherence to the protocol and actual recruitment of consecutive cases will be checked and reported.

### Inclusion and exclusion criteria

Patients presenting to the GP with any new-onset chest pain or pressure (ventral, dorsal and/or lateral), any left or right upper arm pain or any pain in the epigastric, neck or jaw region, at time of presentation not lasting for more than 24 hours, that is considered to be of possible cardiac origin by the GP will be included. Patients presenting burning sensation on the chest not typical for gastric reflux or anxiety with referral to the chest region are also eligible.

Patients presenting other, less specific complaints possibly matching ACS (dyspnoea, nausea, fatigue, etc.) will only be included if at least one of the above mentioned complaints of pain is co-presenting. Patients will be excluded if an obvious emergency is present (haemodynamic instability, otherwise severely ill patient, etc.). Patients will be excluded if symptoms are present for more than 24 hours, if written informed consent is refused during presentation or withdrawn afterwards, if a traumatic cause is present, if complaints are presented that can be regarded as a recurrence of earlier complaints with clear diagnosis (hyperventilation, stable angina) or in case of death of unidentified cause.

### Data collection

Eligible patients will be evaluated by the GP using the following work up schedule:

1. The GP asks written informed consent using a short version of the consent document in the Netherlands or oral informed consent in Belgium.
2. The GP uses the standardised case report form (CRF-HA) for documentation of history and physical examination. If available and if considered as indicated by the GP, an electrocardiogram (ECG) is performed and documented by the GP.
3. The GP is instructed to fill out the presumptive diagnosis together with the decision whether or not to refer to a cardiologist, *before* performing the H-FABP test.
4. The GP obtains one drop of blood from the patient’s finger and starts the PoC H-FABP test. While the test is running, the GP replaces the test in its cardboard package.
5. At this moment, the GP should wait for no less and no longer than 5 minutes before taking the device out of the package again and reading the result. If the GP is still working on point 1–3 as mentioned above in the 5 minutes waiting time, he cannot be influenced by discolouration of the test.
6. Five minutes after starting the test the result is read. The result has to be added to the CRF-HA directly.

By filling out the presumptive diagnosis together with the decision whether or not to refer to a cardiologist on the CRF-HA before reading the result of the PoC H-FABP-test, the regular decision by the GP whether or not to refer to a cardiologist, which is not influenced by the test result, will be formally recorded. Thus, we will be able to compare this regular care with an algorithm using standardised signs and symptoms with PoC H-FABP-testing. This procedure is seen as the next-best option after complete blinding participating GPs for the test result, which is impossible to realise as test results must be read at the point of care within 5 minutes.

GPs will be instructed to base their referral decisions on the current guidelines on ACS, as is common in the Netherlands and Belgium [43]. Participating GPs will be informed that the PoC H-FABP test is currently under study and that the test results should not influence their decision to refer. Only in the few cases that a PoC H-FABP test is positive after an initial decision not to refer, referral policy will be different from usual care since in that rare occasion, the GP is advised to refer. By following the registration work up as mentioned above, we can still conclude that principally, a referral wouldn’t have been made.

Patients that are not referred to secondary care will be instructed and facilitated to have a venous blood sample drawn within the interval between three hours to three days after onset of complaints. Test results will be primarily used to exclude AMI in non-referred patients based on hsTn analysis. However, the GP is instructed to take notice of the result. If the troponin value is elevated, referral to a cardiologist must take place. Furthermore, renal function, based on creatinine and glomerular filtration rate using MDRD-formulas, will be determined. Collected samples will be preserved for a maximum of 15 years for possible future analysis when relevant.

### Final diagnosis

The participating GP will send the CRF-HA (and ECG, if present) to the research team. The research team will contact the GP and, if referral has taken place and if necessary, the hospital, 30 days after inclusion to collect all relevant patient data. Final diagnosis will be established by an expert panel of one independent GP and one independent cardiologist, based on the outcome definitions. If agreement is not reached within the panel, extension will automatically take place to a panel of two cardiologists and two GPs. The expert panel will be blinded to the PoC H-FABP test performed at initial presentation, but will be given access to all other data. This kind of delayed-type reference standard using an expert panel is considered a reasonable alternative, especially in a low-prevalence setting if the definite reference standard (for example, serial troponin measurements or coronary angiography) is reasonably unavailable for reasons of patient risk, cost, etc. [44]. In cases where final diagnosis of our expert panel appears to be different from the clinical diagnosis made by the treating clinicians, we will follow the diagnosis made by our expert panel.

The definitions of cardiac ischaemic situations – ‘stable angina’ (SA), ‘unstable angina’ (UA), ‘acute myocardial infarction’ (AMI) and ‘heart-related sudden death’ – will be standardised for this study based on current literature and have been settled during a panel discussion with two cardiologists, one GP, an ethicist and two researchers before the trial started. SA will be defined as angina pectoris occurring in a patient in predictable situations for months, without a recent change in severity or amount of exertion that is needed for angina pectoris to occur, whereas UA will be defined as new or altered chest pain due to ischaemia without myocardial cell damage reaching a level where significant changes in myocardial damage-markers can be measured [45,46]. AMI will be defined according to the third universal definition of AMI [27]. This definition is mainly based on the combination of clinical evidence and a rise or fall in biomarker values, preferably hsTn. Exact cut-off values for hsTn will depend on the 99th percentile as is widely accepted. Further judgement on whether STEMI (*ST-segment elevation myocardial infarction*) or NSTEMI (*non-ST-segment elevation (non-Q wave) myocardial infarction*) has occurred will depend on the presence of ST-elevations on the ECG.

The classification ‘none of these’ will refer to complaints that are caused by other than cardiac diseases, or by cardiac, non-ischaemic diseases. Those conditions can be described by ‘atypical thoracic complaints’ or, when available, by the clinical diagnosis (e.g. ‘gastric reflux’ , ‘pericarditis’ , etc.). Acute death of identified cause will refer to any case of death occurring in a patient that meets the inclusion criteria for the study and dies after diagnostic evaluation has reached a point where cause of death can reasonably be determined, or death occurring in a patient before evaluation is done but where post-mortem research is performed to identify cause of death. In all other cases of acute death, the outcome will be defined as acute death of unidentified cause.

### Primary and secondary outcome

The most important feature of the algorithm of signs and symptoms combined with PoC H-FABP testing will be to adequately categorise all patients as either positive or negative for ACS. The ability of the algorithm to rule in UA in the ACS group is important, since UA is a condition that does not give a rise in biomarkers and diagnosis depends exclusively on signs and symptoms.

Primary outcome: using multivariate analysis of our data, signs and symptoms that have diagnostic value in an algorithm to predict or exclude ACS and AMI will be identified. The primary outcome measures of the study will be *s*ensitivity, specificity, positive and negative predictive value of an algorithm of those relevant signs and symptoms combined with PoC H-FABP testing for ACS and AMI, in patients with thoracic complaints of new onset in general practice. To determine influence of sex, age, duration of the complaints, and kidney function on clinical performance of H-FABP-testing, subgroup analyses within our study population will be performed [47,48].

Secondary outcome: an economic evaluation by means of an incremental cost-effectiveness ratio (ICER) will be performed. This evaluation will be performed by determination of the ratio between the difference in medical costs and the difference in benefits between the two strategies that are observed in this study, being the usual reference policy of the GP and the reference policy that could be created using a determined algorithm consisting of a clinical score and PoC H-FABP-testing. The benefits will be described as reduction in number of referred patients versus missed diagnoses if the algorithm is used.

### Data management

Data will be stored confidentially and anonymously on the research computer. Researchers obtaining patient data 30 days after study inclusion will work with decoded data. Afterwards, coding will be restored. All data will be held for 15 years after closure of the study. Handling of personal data will comply with the Dutch Personal Data Protection Act and the Belgian privacy legislation (http://wetten.overheid.nl/BWBR0011468/geldigheidsdatum_30-10-2013). Collected blood samples will be preserved for a maximum of 15 years. These samples will only be used for analyses that could contribute to our current field of research.

### Withdrawal or missing data

Should patients refuse informed consent after initial agreement on inclusion, they will be withdrawn from the study and their data abolished. If final diagnosis is impossible due to insufficient data, calculations will be made artificially regarding all patients with missing data as having an ACS, as well as regarding them as having no cardiac ischaemic cause for their initial complaints.

### Sample size and power calculation

An incidence of ACS of 22% would generate 132 patients with ACS and 468 patients without ACS in a study population of 600 patients. To create a usable algorithm consisting of signs, symptoms and H-FABP, the sensitivity of this algorithm should reach 85-90% and specificity should reach 80-85%. In a worst case scenario, where sensitivity would be 85% and specificity would be 80%, combined with the incidence of ACS of 22%, PPV of the algorithm of signs, symptoms and H-FABP for ACS would be 54% (95% C.I. 47-61%) and the NPV 95% (95% C.I. 93-97%). These diagnostic values with their 95% C.I. would be of significant interest, since PPV based on clinical judgment only is 22% and NPV is 95%. Therefore, a sample size of 600 generates adequate precision to find clinically relevant improvement of PPV, as compared to the current situation, and NPV that is at least not diminishing.

### Statistical analyses

Receiver Operating Characteristic (ROC) curves have been used in an earlier stage to find the optimal cut-off point of the venous H-FABP-test [8]. Using 2 × 2 tables and multivariate analyses, including CART analysis, multiple logistic regression, or both, the diagnostic value of the H-FABP-test in combination with clinical findings for ACS and AMI will be assessed [49,50]. Our analysis will result in PPVs and NPVs, sensitivity and specificity and their 95% confidence intervals. Additionally, C-statistics will be presented to quantify overall prediction quality of the models.

### Ethical considerations

The outcome of the test will be subject to this diagnostic study and can therefore have no clinical consequence on the GP’s decision. Patients willing to take part in this study will mainly be treated as usual. Only a finger prick blood analysis for H-FABP will be added to the normal procedure and venous blood sampling will be added in patients that are not referred to secondary care facilities. This study will be in agreement with the current version of the WMA Declaration of Helsinki and will be in accordance with the Dutch Medical Research Involving Human Subjects Act (WMO, http://wetten.overheid.nl/BWBR0009408/geldigheidsdatum_29-10-2013) [51]*.* An independent expert will be available in both countries throughout the study and contact data of this expert will be given to all participating patients. This study protocol has been approved by the ethical review board of Maastricht University for the Netherlands and by the ethical review board of KU Leuven for Belgium. Full research procedures are registered on www.clinicaltrials.gov, NCT01826994.

All GPs including patients for our study will have a liability insurance themselves or via the centre they work for. Maastricht University and KU Leuven have an insurance covering damage to research subjects through injury or death caused by the study, that becomes apparent within 4 years after the end of the study.

### Informed consent

In a short time window of only one consultation, inclusion in our study and performance of the PoC H-FABP test will take place. The majority of the procedure can be categorized as regular care. Therefore the ethical review board was asked to agree on asking oral consent (Belgium) and short written consent (The Netherlands) from patients for taking the PoC H-FABP-test and (if required) the venous blood sample by the GP on initial consultation. This short content contains the four basic principles as stated in article six of the Dutch law for scientific research concerning humans. Subsequently, within one to seven days from initial consultation, patients will be given the opportunity for complete written informed consent after having read an information letter at a more convenient moment. Patients may also decide to withdraw their consent then or at any time thereafter. Only patients who return a written short and complete informed consent will be included in our study.

### Incentives

All GPs will receive €40 per included patient as a compensation for their extra workload.

### Publication policy

All data, results of analyses and conclusions by the study team, either favourable or unfavourable to using PoC H-FABP testing will be disclosed by offering our work for publication in (a) medical journal(s). Our report will follow the international STROBE guideline.

## Discussion

Our study, which commenced in November 2013, will focus on deriving factors to be included in an algorithm of signs and symptoms combined with PoC H-FABP testing (stage 2 of the aforementioned stages of Stiell and Wells) [42]. All criteria for a methodologically correct stage 2 (definition of outcomes and predictor variables, generalisability of subject selection, several statistical and methodological demands) are present in this study, except the inter-observer reliability for the clinical findings, which is difficult to measure in the acute setting in primary care. This study, and subsequent studies aiming to validate, implement and calculate cost-effectiveness (stages 3–6 of Stiell and Wells) in a new clinical setting will possibly enable formulation of a clinical decision rule meeting the current criteria for an effective decision rule [52]. The focus will be on substantially changing clinical behaviour and thus safely reducing the number of referred patients by GPs to secondary care facilities without underlying ACS. An improved triage of patients presenting with chest complaints possibly due to ACS can lead to a substantial cost reduction.

## Abbreviations

ACS: Acute coronary syndrome
AMI: Acute myocardial infarction
GP: General practitioner
H-FABP: Heart-type fatty acid-binding protein
NPV: Negative predictive value
PoC: Point of care
PPV: Positive predictive value
SA: Stable angina
UA: Unstable angina
